# Activation of mucosal immunity as a novel therapeutic strategy for combating brucellosis

**DOI:** 10.3389/fmicb.2022.1018165

**Published:** 2022-12-22

**Authors:** David W. Pascual, Zakia I. Goodwin, Ella Bhagyaraj, Carol Hoffman, Xinghong Yang

**Affiliations:** Department of Infectious Diseases and Immunology, College of Veterinary Medicine, University of Florida, Gainesville, FL, United States

**Keywords:** *Brucella*, mucosal immunity, vaccination, pathogenesis, cell-mediated immunity, CD8^+^ T cells, resident memory T cells

## Abstract

Brucellosis is a disease of livestock that is commonly asymptomatic until an abortion occurs. Disease in humans results from contact of infected livestock or consumption of contaminated milk or meat. *Brucella* zoonosis is primarily caused by one of three species that infect livestock, *Bacillus abortus* in cattle, *B. melitensis* in goats and sheep, and *B. suis* in pigs. To aid in disease prophylaxis, livestock vaccines are available, but are only 70% effective; hence, improved vaccines are needed to mitigate disease, particularly in countries where disease remains pervasive. The absence of knowing which proteins confer complete protection limits development of subunit vaccines. Instead, efforts are focused on developing new and improved live, attenuated *Brucella* vaccines, since these mimic attributes of wild-type *Brucella*, and stimulate host immune, particularly T helper 1-type responses, required for protection. In considering their development, the new mutants must address *Brucella*’s defense mechanisms normally active to circumvent host immune detection. Vaccination approaches should also consider mode and route of delivery since disease transmission among livestock and humans is believed to occur *via* the naso-oropharyngeal tissues. By arming the host’s mucosal immune defenses with resident memory T cells (TRMs) and by expanding the sources of IFN-γ, brucellae dissemination from the site of infection to systemic tissues can be prevented. In this review, points of discussion focus on understanding the various immune mechanisms involved in disease progression and which immune players are important in fighting disease.

## Introduction

Brucellosis remains a worldwide problem ranking third among eight neglected zoonotic diseases (Mableson et al., 2014), and is the most common zoonotic disease worldwide (Corbel, 1997; Godfroid et al., 2005). In humans, brucellosis is generally results from the consumption of unpasteurized dairy products or exposure to aerosols from infected livestock (Spink, 1956; Foley et al., 1970; Centers for Disease Control (CDC), 1983; Young, 1983; Chomel et al., 1994; Malik, 1997; Troy et al., 2005; de Figueiredo et al., 2015). In livestock, brucellosis was originally believed to be solely a sexually transmitted disease resulting in fetal abortion (Poester et al., 2013; Olsen and Palmer, 2014; de Figueiredo et al., 2015), but oropharyngeal infection is deemed to be the probable mode of transmission following exposure to an aborted fetus or birthing tissues (Olsen and Palmer, 2014; Cotterill et al., 2018). In fact, one study found that *Brucella*-infected bovine umbilicus contained 2 × 10^8^ to 4 × 10^9^ CFUs/g and bovine fetal cotyledons, 5 × 10^11^ to 1 × 10^13^ CFUs/g tissue (Alexander et al., 1981). Thus, brucellae can concentrate to high numbers, and following abortion, the placenta and fetus can expose herd members to high numbers of brucellae. On the human side, this Gram-negative species is highly infectious with less than 100 CFUs needed for blood borne exposure (Pappas et al., 2006) or less than 2,000 CFUs for pulmonary infection (Teske et al., 2011; Henning et al., 2012). Regardless of the route of infection, brucellae can disseminate systemically resulting in flu-like symptoms (Young, 1983; Malik, 1997; Troy et al., 2005; de Figueiredo et al., 2015). Since current vaccines are not completely protective in livestock, more efficacious brucellosis vaccines are needed to protect livestock and to prevent zoonosis. Vaccines for humans would also aid in protecting livestock producers.

This review considers alternate routes of vaccination, specifically, mucosal routes, specifically in the context of *Brucella* prophylaxis. Because natural infection generally occurs *via* crossing of the mucosal barrier, vaccination *via* this route should improve vaccine efficacy by mounting both local and distal responses of effector and memory B and T cells. While *Brucella* vaccination is often administered parentally in cattle and wildlife, mucosal vaccination *via* the conjunctival route is practiced in sheep as a strategy to reduce vaccine-induced abortions. While the T cell response is considered crucial to the *Brucella* vaccine-induced protective response, few studies have examined the mucosal T cell responses in ruminant species following conjunctival vaccination. Similarly, most vaccine studies in experimental animal models focus on the systemic response following parenteral vaccination. Thus, a void exists between the understanding host immunity and the routes of vaccination particularly in natural hosts. Past research in livestock species have focused on protection against abortion, serological responses, and peripheral blood T cell responses. More evaluations are needed in both natural hosts and laboratory animal models in determining additional parameters of protection against both abortion and infection. Infection prevention is particularly relevant when considering vaccine development for humans.

To meet this objective, the review first explores a historical perspective of brucellosis and its prevalence through the course of time followed by the etiology of this disease. We then describe current knowledge of host immunity to brucellosis, examining various cell-mediated immunity parameters that correlate with protection. A brief description of currently available vaccines is provided. With this background information, we move to studies examining mucosal routes of vaccination. We conclude describing the potential benefits of mucosal vaccination in both animal and human hosts.

## Historical perspective

Brucellosis is believed to have been problematic for humans for at least several millennia or maybe longer dating to the domestication of goats and sheep. Some of the first suggestions of brucellosis infections in humans date back about 3,300 years, whereby a possible *Brucella* peptide signature was identified in cheese remnants found in Egyptian tombs (Greco et al., 2018). In addition, lesions in human vertebrae remains from Early Bronze Age and the Pompeii volcanic eruption in 79 AD are suggestive of *Brucella* infection (D'Anastasio et al., 2011). Not until 1887 was the etiological agent responsible for brucellosis discovered by the British surgeon, David Bruce, while serving in Malta. He first identified small coccobacilli causing “Malta Fever,” when isolated from the spleen of a victim (Bruce, 1889; Vassallo, 1992), and called it *Micrococcus melitensis* (Bruce, 1887). After its discovery in goats’ blood and later in their milk, consumption of goats’ milk was surmised to be the source of Malta Fever transmission. This suspicion was corroborated when the British military prohibited goat milk consumption by its Malta personnel, resulting in a dramatic decline in disease incidence (Vassallo, 1992). In 1895, the Danish veterinary pathologist, L. F. Benhard Bang, discovered the related, *Bacillus abortus*, responsible for abortion in cattle, and upon subsequent infection of an isolate, could induce abortion in cattle, sheep, and goats fulfilling Koch’s postulates (Bang, 1897; Bang, 1906). Based on the observation that both *Bacillus abortus* and *Micrococcus melitensis* induced abortion, presence in milk, and shared morphology and seroreactivity, Evans concluded these species were related (Evans, 1918). In 1920, in honor of Dr. Bruce, the three related species of *M. melitensis*, *B. abortus*, and *B. suis* were named in the new genus, *Brucella* (Vassallo, 1992).

## *Brucella* etiology

### *Brucella*, the pathogen

The Gram-negative *Brucella* species are highly homogeneous sharing more than 94% DNA homology (Wattam et al., 2009; Van der Henst et al., 2013; Whatmore and Foster, 2021), and 12 species have been identified as animal and human pathogens (Olsen and Palmer, 2014; Roop et al., 2021; Whatmore and Foster, 2021). Their genomes are composed of two circular chromosomes (Wattam et al., 2009; Roop et al., 2021). *Brucella* species are ubiquitous, infecting both land and marine animals (Guzmán-Verri et al., 2012; Roop et al., 2021; Whatmore and Foster, 2021). Three species are the primary cause of disease in livestock and can be problematic for humans due to incidental exposures. *Brucella melitensis* causes disease primarily in goats and sheep (Poester et al., 2013; Olsen and Palmer, 2014). *Bacillus abortus* is primarily a disease of cattle, but has been introduced into wildlife both in the United States and other countries (Poester et al., 2013; Olsen and Palmer, 2014). *Brucella suis* is primarily a disease of domestic and feral pigs (Corbel, 1997; Poester et al., 2013; Roop et al., 2021). Each of these is the primary cause of disease in humans, and the remaining 8 species are found in specific hosts (Wattam et al., 2009; Roop et al., 2021; Whatmore and Foster, 2021). However, opportunistic species have been reported in limited or rare human infections including, *B. inopinata* (Scholz et al., 2010; Roop et al., 2021), the amphibian *B. inopinata*-like (Rouzic et al., 2021), and *B. canis* (Roop et al., 2021). Recent genomic evidence suggests that a number of *Ochrobactrum* species as being related to *Brucella*, which would dramatically expand this genus (Hördt et al., 2020; Moreno et al., 2022).

### Livestock disease

The four major *Brucella* pathogens responsible for livestock disease (Poester et al., 2013; Olsen and Palmer, 2014; Goodwin and Pascual, 2016) are *B. abortus*, *B. melitensis*, *B. suis*, and *B. ovis*, and each cause abortion (Bang, 1897; Godfroid et al., 2011; Poester et al., 2013; Byndloss and Tsolis, 2016). The absence of symptoms prior to abortion is problematic. Brucellosis is a chronic disease with possible swelling of lymph nodes near sites of infection. The most common clinical manifestation of animal brucellosis is reproductive loss resulting from abortion, birth of weak offspring, or infertility (Poester et al., 2013; Olsen and Palmer, 2014; Goodwin and Pascual, 2016; Rossetti et al., 2022). *Brucella abortus* localizes and replicates within the rough endoplasmic reticulum of trophoblastic epithelial cells in pregnant ruminants (Meador and Deyoe, 1989). However, placental infections frequently cause abortions after infection, particularly for the first offspring, and reduce fertility (Enright, 1990; Olsen and Palmer, 2014). Fetal pneumonia and necrotizing placentitis are implicated as the cause of abortion (Poester et al., 2013; Olsen and Palmer, 2014). Exposure to the aborted fetus and placenta is likely responsible for the persistence of *B. abortus*, *B. melitensis*, and *B. suis* in herds and populations. Though not zoonotic, *B. ovis*, is responsible for disease primarily in sheep, but is limited to mostly rams and transiently infect ewes (Ridler and West, 2011; Olsen and Palmer, 2014; Rossetti et al., 2022). *Brucella ovis* can cause epididymitis, which can result in infertility, and its presence in semen can be detected in rams with or without epididymitis (Ridler and West, 2011; Olsen and Palmer, 2014; Rossetti et al., 2022).

### Human disease

Human brucellosis can be a debilitating disease, especially if untreated (Pappas et al., 2005, 2006; Franco et al., 2007; Cross et al., 2019). Brucellosis poses an occupational hazard common to abattoir workers, or by needle-stick by laboratory workers and veterinarians administering live brucellosis vaccines (Buswell et al., 2016; Pereira et al., 2020). Human brucellosis is more often acquired subsequent to consumption of unpasteurized dairy products (Chomel et al., 1994; Pappas et al., 2005; Baldi and Giambartolomei, 2013; Dadar et al., 2019; Adetunji et al., 2020). The American Academy of Pediatrics recommends against the consumption of unpasteurized milk by pregnant women and children (American Academy of Pediatrics, 2014). Brucellosis prevails along the Mediterranean rim, Middle East, Central Asia, South America, and the United States bordering Mexico (Chomel et al., 1994; Malik, 1997; Pappas et al., 2005; Corbel, 2006; Pappas et al., 2006). Its presence is attributed mostly to the inability to rid disease from livestock. The enormous cost of brucellosis to the livestock industry, as well as its impact on public health, has prompted many countries to adopt brucellosis control and eradication programs (Olsen and Stoffregen, 2005). In the United States, a brucellosis eradication program was established in 1954 aiding in the elimination of *B. abortus* infections from cattle. Vaccination of heifers using *B. abortus* strain 19 (S19), then subsequently with RB51, has been practiced to reduce the incidence of the disease and prevent *B. abortus*-induced abortions (Schurig et al., 2002). Ridding brucellosis from animal herds and pasteurization of dairy products reduce disease in humans.

The incidence of disease varies among the endemic regions (Pappas et al., 2006; Franco et al., 2007), but actual case numbers may be higher by as much as 26-fold due to misdiagnosis and underreporting (Franco et al., 2007; Hull and Schumaker, 2018). The high incidence of *Brucella* infections is attributed to the sustained prevalence of brucellosis in infected livestock (Young, 1983), that are the source of unpasteurized milk consumed in various dairy products (Pappas et al., 2005; Baldi and Giambartolomei, 2013; Dadar et al., 2019; Adetunji et al., 2020). In addition, a number of cases have been attributed to an aerosol exposure from *Brucella*-infected livestock (Corbel, 1997), laboratory acquired (Traxler et al., 2013), or an accidental biopharmaceutical release (Pappas, 2022). In the latter case, more than 10,000 individuals became infected with brucellosis (Pappas, 2022). However acquired, brucellosis is seldom (<0.5% of cases) life-threatening in humans (Ariza et al., 1995; Corbel, 1997), but human abortions occur, though rare [rev. in (Arenas-Gamboa et al., 2016)]. Acute disease manifests with flu-like symptoms such as fever, chills, malaise, headaches, with the presence of hepatomegaly and splenomegaly (Corbel, 1997; Pappas et al., 2005; Corbel, 2006; Franco et al., 2007). Despite rigorous antibiotic treatment, brucellosis can progress to a chronic disease exhibiting symptoms of relapsing undulant fever, protracted fatigue, and malaise (Young, 1983; Ariza et al., 1995; Franco et al., 2007; Baldi and Giambartolomei, 2013; Hull and Schumaker, 2018), and have positive *Brucella* blood cultures (Ariza et al., 1995; Corbel, 1997). Patients can further develop neurological complications, endocarditis, or arthritis (Rajapakse, 1995; Reguera et al., 2003; Shen, 2008; Adetunji et al., 2018; Lacey et al., 2018). Although *Brucella* is sensitive to antibiotics *via* a prolonged two-antibiotic regimen (Ariza et al., 1995; Corbel, 1997), sequelae can still remain in ~16% of the infected individuals (Ariza et al., 1995), of which 50% remain bacteremic (Ariza et al., 1995). Mucosal infection is the most likely means of brucellosis transmission, yet the absence of symptoms or pathology in the intestinal tract (Ariza et al., 1995; Ablin et al., 1997; Ron-Román et al., 2014) suggests that brucellae rapidly transverse the intestinal tract, local phagocytic cells become infected and leave this tissue, or that other mucosal sites are sensitive to infection. Oropharyngeal tissues are the most likely site of infection since pharyngitis is often observed (Carpenter and Boak, 1932; Poelma and Pickens, 1932; Suraud et al., 2007; Zachou et al., 2008; Ron-Román et al., 2014), and cervical lymph nodes are selectively infected (von Bargen et al., 2015; Rouzic et al., 2021). Treatment requires a combination therapy of antibiotics for 6 weeks generally involving doxycycline + streptomycin, doxycycline + gentamicin, or doxycycline + rifampin (Ariza et al., 2007; Bosilkovski et al., 2021).

## *Brucella* immunology

### *Brucella*, a stealth pathogen

As a means to sustain intracellular survival, *Brucella* has evolved a number of mechanisms to avoid host recognition and establish infection (Celli et al., 2019; Roop et al., 2021). Following bacteremia, macrophages are one of the principal cells targeted by *Brucella* to sustain infection (Gomes et al., 2012; Celli et al., 2019; Bhagyaraj et al., 2021). Once brucellae achieve intracellular infection, their elimination proves to be more difficult as these have a number of tools to evade the host immune system. As such, brucellae exist in *Brucella*-containing vacuoles (BCVs; Gomes et al., 2012; Celli et al., 2019; Roop et al., 2021), which traffic in the endocytic pathway incorporating endosomal membrane proteins, e.g., calreticulin and calnexin1, avoiding phagolysosome maturation and killing (Pizarro-Cerdá et al., 1998; Celli et al., 2003; Starr et al., 2008; Gomes et al., 2012; Celli et al., 2019; Bhagyaraj et al., 2021; Roop et al., 2021) in a VirB-dependent fashion (Celli et al., 2003; Starr et al., 2008; Gomes et al., 2012; Roop et al., 2021). To avoid TLR4 and other LPS-sensitive innate detection sensors, *Brucella* expresses a low endotoxic LPS (Forestier et al., 2000; Martirosyan et al., 2011; Conde-Álvarez et al., 2012; Byndloss and Tsolis, 2016). *Brucella* can also infect dendritic cells (Bhagyaraj et al., 2021), and suppress dendritic cell maturation (Salcedo et al., 2008). To interfere with TLR2 and TLR4 signaling, *Brucella* produces TcpB, an analog for mammalian Toll/interleukin 1 receptor (TIR) domain-containing adaptor protein (TIRAP), to suppress NF-κB activation and cytokine secretion (Alaidarous et al., 2014; Snyder et al., 2014). TcpB can also degrade caspases 1, 4, and 11, and ultimately suppress IL-1 production (Jakka et al., 2017). To minimize host adaptive immune responses, *Brucella* has the capacity to interrupt antigen presentation *via* inhibition of MHC class I (Barrionuevo et al., 2013; Velásquez et al., 2017; Barrionuevo and Giambartolomei, 2019) and class II molecules (Barrionuevo et al., 2008; Velásquez et al., 2017; Barrionuevo and Giambartolomei, 2019; Milillo et al., 2019).

Type 1 IFNs are often deemed important for anti-viral defense (McNab et al., 2015; Takaoka and Yamada, 2019), and few studies have considered the role of type 1 IFNs following infection with wild-type (wt) *Brucella* (de Almeida et al., 2011; Gorvel et al., 2014; Khan et al., 2016; Costa Franco et al., 2018; Guimarães et al., 2019). *Brucella* infection interferes with monocytic DC maturation (Gorvel et al., 2014). The induction of the type 1 IFN pathway was found to be sensing stimulator of interferon genes (STING)-dependent evidenced by the recognition of both *Brucella*’s DNA (de Almeida et al., 2011; Costa Franco et al., 2018) and c-di-GMP (Khan et al., 2016; Costa Franco et al., 2018; Guimarães et al., 2019). Wt *Brucella* showed enhanced splenic colonization in STING^−/−^ mice (Costa Franco et al., 2018; Khan et al., 2020) supporting the notion that wt *B. melitensis* can suppress STING early in infection.

### Th1 cell immunity and brucellosis

Cellular immunity is essential for protection against brucellosis (Table 1). An inflammatory or T helper (Th)1 cell response is required to eliminate brucellae in an IL-12- (Zhan and Cheers, 1995) and TNF-α-dependent manner (Zhan and Cheers, 1998) for the stimulation of IFN-γ. As a result, protection to *Brucella* is abated in IFN-γ^−/−^ mice (Murphy et al., 2001; Skyberg et al., 2012) further supporting the importance of IFN-γ to protection. Yet, there may exist alternative or cooperative pathways of protection. One critical observation is that the degree of susceptibility in IFN-γ^−/−^ mice varied between C57BL/6 (B6) and BALB/c backgrounds. B6 IFN-γ^−/−^ mice are more highly susceptible to death sooner than BALB/c IFN-γ^−/−^ mice (Murphy et al., 2001; Skyberg et al., 2012). Moreover, vaccination of BALB/c IFN-γ^−/−^ mice with Δ*znuA B. melitensis* mutant did show reduced brucellae colonization from challenge suggesting other mechanisms that can contribute to immune protection (Clapp et al., 2011, 2016), and these vaccinated IFN-γ^−/−^ mice did not show increased susceptibility to death. Both strains of IFN-γ^−/−^ mice showed increased susceptibility to osteoarthritis (Skyberg et al., 2012; Lacey et al., 2016). In fact, *Brucella*-infected patients with reduced IFN-γ capacity showed increased sensitivity to osteoarticular complications (Rafiei et al., 2006; Hedayatizadeh-Omran et al., 2010).

**Table 1:** Both route and *Brucella* strain used for vaccination influence T cell responses.

*Brucella* strain used to vaccinate^1^	Route^2^	Tissue^3^	Induced T cell predilection	Induced memory T cell subset^4^	References
Wt *Bacillus abortus* 2308	IP	Spleen	CD4^+^	ND	Dornand et al. (2004)
*B. abortus* S19	IV	Spleen	CD4^+^	ND	Araya et al. (1989)
*B. abortus* S19	IP	Spleen	CD4^+^	ND	Zhan and Cheers (1995)
*B. abortus* RB51	IP	Spleen	CD4^+^	ND	Yang et al. (2016) and He et al. (2001)
*B. abortus* RB51	IT	Spleen	CD4^+^	CD44^+^	Muñoz et al. (2008)
*B. abortus* RB51	IN	Spleen	CD4^+^	CD44^+^	Muñoz et al. (2008)
*B. abortus* RB51	IN	Lung, spleen, LN	CD8^+^	ND	Clapp et al. (2016)
Δ*znuA* Δ*norD B. abortus-lacZ*	IP	Spleen	CD4^+^	ND	Yang et al. (2016)
Δ*znuA* Δ*norD B. abortus-lacZ*	oral + IN	Lung, spleen	CD8^+^	TRMs	Wang et al. (2020)
*B. abortus* RB51	oral + IN	Lung, spleen	CD4^+^	TRMs	Wang et al. (2020)
Wt *Brucella melitensis* 16M	IP	Spleen	CD4^+^	ND	Vitry et al. (2012)
Wt *B. melitensis* 16M + antibiotic	IP	Spleen	CD4^+^	ND	Vitry et al. (2014) and Hanot Mambres et al. (2016)
Wt *B. melitensis* 16M + antibiotic	IN	Lung, spleen	CD4^+^, CD8^+^	ND	Hanot Mambres et al. (2016)
Δ*znuA B. melitensis*	IN	Lung, spleen, LN	CD8^+^	TEMs, TCMs	Clapp et al. (2016)
*B. melitensis* Rev. 1	IN	Lung, spleen	CD8^+^	ND	Clapp et al. (2016)
*B. melitensis* WR201	oral	Lung, spleen	CD4^+^	ND	Yingst et al. (2013)
Δ*znuA* Δ*norD B. melitensis*-mCherry	oral + IN	Lung, spleen	CD4^+^ and CD8^+^	TEMs, TCMs, TRMs	Goodwin et al. (2022)
*B. melitensis* Rev. 1	oral + IN	Lung, spleen	CD4^+^	TEMs, TCMs, TRMs	Goodwin et al. (2022)

1 Various strains of *Brucella* have been tested including wild-type (Wt), strain 19 (S19), RB51, Rev 1 vaccines, and different defined mutants.

2 IP, intraperitoneal; IV, intravenous; IT, intratracheal; IN, intranasal.

3 Tissue examined for T cell responses.

4 ND, not determined; TCMs, central memory T cells; TEMs, effector memory T cells; TRMs, resident memory T cells.

Essential sources of IFN-γ include CD4^+^ (Murphy et al., 2001; Vitry et al., 2012; Yingst et al., 2013; Vitry et al., 2014; Yang et al., 2016), CD8^+^ T cells (Clapp et al., 2011; Durward-Diioia et al., 2015; Clapp et al., 2016; Yang et al., 2016; Wang et al., 2020), or both (Araya et al., 1989; Hanot Mambres et al., 2016; Goodwin et al., 2022). NK cells also provide IFN-γ to activate macrophages and DCs (Dornand et al., 2004; Bhagyaraj et al., 2021). The role of CD4^+^ and CD8^+^ T cells has been extensively studied to learn correlates of protection against brucellosis (Table 1). How IFN-γ-producing T cells are elicited is dependent upon the *Brucella* mutant or vaccine strain used, as well as, its mode of delivery for vaccination (Table 1). Clearly, CD4^+^ Th1 cells provide a primary source of IFN-γ to combat *Brucella* infection (Zhan et al., 1995; He et al., 2001; Vitry et al., 2012; Yingst et al., 2013; Vitry et al., 2014), and vaccination by the parenteral route with livestock vaccines elicited mostly CD4^+^ T cell-dependent responses (Table 1). In one study, protection was deemed CD4^+^ T cell-dependent since orally vaccinated CD8^−/−^ mice with a purine auxotrophic *B. melitensis* mutant were not protected against virulent *B. melitensis* challenge (Yingst et al., 2013). In a separate study, vaccination of MHC class II^−/−^ mice was accomplished using a low-dose parenteral infection with wt *B. melitensis* 16M followed by antibiotic treatment. When challenged with wt *B. melitensis* 16M, these mice showed elevated splenic brucellae burden and reduced IFN-γ-producing cells when compared to similarly vaccinated and challenged immunocompetent mice (Vitry et al., 2014). In a subsequent study (Hanot Mambres et al., 2016), a similar strategy was applied with a nasal wt *B. melitensis* 16M infection of MHC class II^−/−^ and TAP1^−/−^ mice followed by antibiotic treatment prior to nasal challenge with wt *B. melitensis* 16M. Both MHC class II^−/−^ and TAP1^−/−^ mice showed equivalent protection to similarly immunized and challenged immunocompetent mice. The investigators concluded that the source of IFN-γ-producing cells can be either CD4^+^ or CD8^+^ T cells for conferring protection. Such results corroborate findings from an earlier study where adoptive transfer of immune CD4^+^ or CD8^+^ T cells from *B. abortus* S19-vaccinated mice reduced splenic brucellae colonization following challenge of recipients with wt *B. abortus* (Araya et al., 1989). Thus, based upon these findings, dependence on CD4^+^ T cell immunity for protection against virulent *Brucella* challenge is influenced by the source of the immunizing *Brucella* strain used (Table 1).

### CD8^+^ T cell immunity and brucellosis

The lack of memory CD8^+^ T cells has been suggested as a means for enabling brucellae persistence (Durward-Diioia et al., 2015). In contrast, others suggest that CD8^+^ T cell immunity is expandable for brucellosis (Vitry et al., 2012; Yingst et al., 2013; Vitry et al., 2014). Only a few studies have investigated the role of CD8^+^ T cells in immunity to *Brucella* infections (Table 1). One study focused on using mice deficient of their immunoproteasome (lacking β1i, β2i, and β5i subunits) to minimize MHC class I antigen presentation. Infection of these mice with wt *B. abortus* 2308 resulted in nearly complete loss of IFN-γ^+^ CD8^+^ T cells, as well as, a substantive reduction in IFN-γ^+^ CD4^+^ T cells (Guimarães et al., 2018). Such evidence points to CD8^+^ T cells contributing to IFN-γ generation. CD8^+^ T cell immunity may also be influenced by targeted mutations made in *Brucella* to attenuate its infection. One notable mutation was the deletion of *znuA*, a gene involved in zinc uptake, as a means to inactivate zinc-dependent enzymes in *Brucella*, particularly its superoxide dismutase. The Δ*znuA B. abortus* mutant exhibited diminished growth capacity in macrophages and rendered protective qualities, when administered parenterally, similar to conventional *B. abortus* S19 and RB51 vaccines (Yang et al., 2006).

The route of vaccination may also influence the T cell response elicited (Table 1). Adapting this same mutation in *B. melitensis* resulted in an attenuation signature similar to that of Δ*znuA B. abortus*. Nasal vaccination with the Δ*znuA B. melitensis* mutant showed a T cell bias noted by the stimulation of IFN-γ^+^ CD8^+^ T cells than by CD4^+^ T cells (Clapp et al., 2016). Such attribute posed an interesting question of whether the preferential stimulation of CD8^+^ T cells was due to the mutation or the mode of vaccination. To test this question, a second mutation was introduced into Δ*znuA B. abortus*. The *norD* gene, which encodes for nitric oxide reductase was selected since it showed a modest reduction in virulence as a single gene deletion in *B. suis* (Loisel-Meyer et al., 2006). The development of the double *ΔznuA* Δ*norD B. abortus* mutant carrying the *lacZ* reporter gene (znBAZ) prompted testing *via* parenteral immunization resulting in the stimulation of both IFN-γ-producing and polyfunctional CD4^+^ and CD8^+^ T cells (Yang et al., 2016). Yet, the numbers of splenic IFN-γ^+^ and polyfunctional CD4^+^ T cells exceeded the counterparts for CD8^+^ T cells.

To learn how these mutations in *Brucella* may influence the types of T cell responses elicited following mucosal vaccination (Table 1), an oral prime, nasal boost vaccination regimen was devised for znBAZ (Wang et al., 2020). The notable attribute that distinguished znBAZ’s immunogenicity was the number of IFN-γ^+^ CD8^+^ and polyfunctional CD8^+^ T cells doubled those of IFN-γ^+^ CD4^+^ and polyfunctional CD4^+^ T cells present in the lungs. Similarly primed, boosted mice with RB51 showed no bias for IFN-γ^+^ CD8^+^ T cells, mostly being IFN-γ^+^ CD4^+^ T cells (Wang et al., 2020). Another key attribute found with orally primed, nasally boosted mice with znBAZ is the induction of resident memory T cells (TRMs; Table 1). TRMs are distinguished by their expression of memory markers, CD44^high^ L-selectin^−^ CD69^+^ and either CD103^+^ or CD103^−^, and reside in the mucosal epithelium (Turner et al., 2013; Mueller and Mackay, 2016; Szabo et al., 2019). The mucosal epithelium is where exposure to wt *Brucella* is most likely to occur; hence, the need to arm the epithelium with memory T cells is deemed essential to eradicate *Brucella*-infected cells. As a result, both CD4^+^ and CD8^+^ TRMs were elicited to greater levels subsequent to znBAZ vaccination than the level seen in those mice similarly vaccinated with RB51 (Wang et al., 2020). In a similar vein, the same genetic mutations were performed to generate the *ΔznuA* Δ*norD B. melitensis* mutant carrying the mCherry reporter gene (znBM-mC; Goodwin et al., 2022). As accomplished with znBAZ vaccination, CD4^+^ and CD8^+^ TRMs were induced in the lungs of mice orally primed, nasally boosted with znBM-mC (Table 1), but TEMs and TCMs were also detected (Goodwin et al., 2022). This evidence suggests that the mode of vaccination can contribute to types of IFN-γ^+^ and polyfunctional T cells, as well as to the TRMs induced, but cannot diminish the influence of the *Brucella*’s mutations used to develop these strains.

### Th17 cell immunity and brucellosis

As suggested above, alternative immune players may contribute to protection against *Brucella* infections. One such possibility examined is the role of a different proinflammatory cytokine, IL-17. IL-17, important for protection against various extracellular mucosal pathogens, is derived from innate and adaptive lymphocytes (Mills, 2022). IL-17 is involved in neutrophil recruitment and promotes IL-22 and antimicrobial peptide production (Mills, 2022). Although often overlooked, IL-17 has had varied impact on protection against brucellosis, but IL-17 may be more relevant upon extracellular brucellae release from killed host cells. For example, mice vaccinated with Δ*znuA Brucella* mutants and treated *in vivo* with an anti-IL-17 antibody (Ab) showed significant increases in colonization suggesting a role for IL-17 and protection (Clapp et al., 2011; Pasquevich et al., 2011; Clapp et al., 2016). Although IL-17 production is augmented in mucosally vaccinated IFN-γ^−/−^ mice (Clapp et al., 2011, 2016), IL-17 production is not linked to the development of osteoarthritis in *Brucella*-infected IFN-γ^−/−^ mice (Skyberg et al., 2012). In contrast, oral prime, nasal boost with znBAZ did elicit IL-17^+^ CD4^+^ and CD8^+^ TRMs, but *in vivo* neutralization of IL-17 during the post-challenge phase failed to reverse znBAZ’s protective qualities, i.e., the diminished lung and splenic colonization by wt *B. abortus* 2308 remained intact (Wang et al., 2020). However, the spleens from IL-17^−/−^ mice that were orally primed, nasally boosted with znBM-mC showed increased brucellae colonization following pulmonary challenge with wt *B. melitensis* 16M suggesting that IL-17 may be important for maintaining systemic protection (Goodwin et al., 2022). Nasal infection with virulent *B. melitensis* 16M minimally impacted brucellae colonization of the lungs and spleen 4 weeks post-infection of IL-17RA^−/−^ and IL-23p19^−/−^ mice, but did enable greater colonization of the lungs at 5 and 12 days post-infection of IL-17RA^−/−^ mice. This suggests IL-17’s role may be more relevant early in infection (Hanot Mambres et al., 2016), possibly tied to neutrophil recruitment. IL-17 derived from γδ T cells is also thought to be important early in *B. abortus* infection (Skyberg et al., 2011).

Th17 cells have been reported to produce GM-CSF and IL-22 following RORγt activation (Liang et al., 2006; Zheng et al., 2007; Codarri et al., 2011; El-Behi et al., 2011). Some consider IL-22-producing T cells as a separate entity, e.g., Th22 cells (Dudakov et al., 2015), since Runx1 has been recently found to coactivate RORγt (Sekimata et al., 2019). The stimulation of IL-22 is associated with protection of the mucosal epithelium by enhancing the epithelial barrier and increasing defensins production (Valeri and Raffatellu, 2016), and is responsible for stimulation of the heme scavenger, hemopexin, to sequester iron from bacteria (Sakamoto et al., 2017). Examination of IL-22’s role was pursued subsequent mucosal vaccination with *ΔznuA B. melitensis*, and IL-22 production was found to be augmented in both immunocompetent and IFN-γ^−/−^ mice (Clapp et al., 2011, 2016). For nasally *ΔznuA B. melitensis*-vaccinated mice, IL-22 was mostly derived from CD8^+^ T cells (Clapp et al., 2016). Another consideration for IL-22’s relevance is the observed lack of intestinal infection by *Brucella* (Ariza et al., 1995; Ablin et al., 1997). IL-22 production was augmented following oral *ΔznuA B. melitensis* vaccination (Clapp et al., 2011). Oral infection remains to be tested in IL-22^−/−^ mice to determine if increased intestinal pathology occurs because of a reduced barrier. However, IL-22^−/−^ mice infected parenterally with virulent *B. melitensis* 16M showed only a minimal impact early in infection with a modest reduction in IFN-γ^+^ CD4^+^ T cells (Vitry et al., 2012).

### γδ T cells and brucellosis

γδ T cells serve as sentinels in mucosal surfaces and respond to infectious agents *via* their variable TCRs and pathogen recognition receptors (Holderness et al., 2013). These cells are an important source of IL-17 (Agerholm and Bekiaris, 2021), but also responsible for the production of other proinflammatory cytokines including IFN-γ (Skyberg et al., 2011; Demars et al., 2019). γδ T cells contribute to early innate cell activation noted by the increased brucellae burden following infection of TCRδ-deficient mice with wt *B. abortus* 2308 (Skyberg et al., 2011) or wt *B. melitensis* 16M (Hanot Mambres et al., 2016). Consistent with the notion of early involvement, macrophages in the presence of γδ T cells benefitted by the enhanced clearance of infecting brucellae (Skyberg et al., 2011). However, γδ T cells’ impact is not relevant during the late phase of infection evidenced by the lack of differences in brucellae tissue burdens when compared to similarly infected wt mice (Skyberg et al., 2011; Hanot Mambres et al., 2016). Instead, γδ T cells’ contribution to brucellae clearance from tissues may be dependent upon route of exposure (Skyberg et al., 2011; Hanot Mambres et al., 2016; Demars et al., 2019). TCRδ-deficient mice given an intradermal infection with wt *B. melitensis* 16M showed no difference in brucellae tissue burdens from wt mice at early or late time points (Demars et al., 2019), suggesting that peripheral γδ T cells may be less impacted by *Brucella* infections than mucosal γδ T cells (Hanot Mambres et al., 2016).

### Livestock T cell immunity

The above studies focused primarily on results obtained in mice to learn relevant correlates of protection. Such studies are often more difficult to conduct in the natural host due to limitations in readily available genetic models to test various hypotheses. Nonetheless, significant knowledge can still be extrapolated from rodent studies and *in vitro* studies using livestock lymphocytes. Surprisingly, only a few studies have delved into studying IFN-γ-producing T cell responses in livestock following infection with wt *Brucella* or subsequent vaccination. These studies also varied in their mode of *Brucella* delivery. Additionally, studies that did investigate host T cell responses mostly relied upon peripheral blood, not tissue-derived T cells. The limitation of peripheral blood T cell responses is that these lymphocytes are ever evolving, and timing is critical to capture the moment of optimal responsiveness. As in the case of one study, cows were conjunctivally infected with *B. suis* or *B. abortus*, and their peripheral blood CD4^+^ T cells were shown to produce IFN-γ following *in vitro* Ag restimulation using Brucellergene®, (a preparation of cytoplasmic proteins derived from a rough mutant of *B. melitensis*; Weynants et al., 1998), Little IFN-γ production was detected for CD8^+^ or γδ T cells (Weynants et al., 1998). However, in a separate study, bovine γδ T cells could aid in activating macrophages *in vitro via* IFN-γ, suggesting that γδ T cells may be more important early during the infection if sufficiently activated (Skyberg et al., 2011). Another study investigated IFN-γ production by peripheral blood T cells from intramuscular (IM) RB51-vaccinated heifers, and following Ag restimulation, the induced IFN-γ was mostly derived from CD4^+^ T cells (Boggiatto et al., 2020). However, one study did pursue an evaluation of memory T cells (up to one and one-half years) and various cytokine responses by peripheral blood T cells from subcutaneously (SC) RB51-vaccinated calves (Dorneles et al., 2015). These heifers were dosed twice with RB51 one year apart. While both CD4^+^ and CD8^+^ T cells showed increased cytokine production following *in vitro* Ag restimulation, the majority of the IFN-γ and IL-17 came from CD4^+^ T cells (Dorneles et al., 2015). Granzyme B^+^ and perforin^+^ CD8^+^ T cells were also augmented subsequent the RB51 boost.

An in-depth immune analysis was performed following conjunctival infection with wt *B. melitensis* H38S of sheep (Suraud et al., 2008). Brucellae colonization of various mucosal sites including nasal and eye secretions, eyelids and lacrimal glands, tonsils, various head and neck lymph nodes (HNLNs), and distal mesenteric LNs, precapsular LNs, and spleen were monitored up to 4 weeks post-infection. Increases in IFN-γ production following *Brucella* Ag-restimulation of regional and distal LN lymphocytes were observed (Suraud et al., 2008). A separate study examined T cell profiles following variable SC dosing of sheep with Rev. 1 vaccine, and found elevated CD4^+^ and CD8^+^ T cell levels, but did not discern whether these CD8^+^ T cells included γδ T cells (Curina et al., 2018). Total memory T cells were elevated, but did not distinguish between CD4^+^ and CD8^+^ T cells. Another study measured T cell responses to bp26 and Omp31 peptides in sheep SC vaccinated thrice with *B. melitensis* M5-90 vaccine, and found elevated IFN-γ responses mostly derived from CD4^+^ T cells (Wang et al., 2014). The investigators speculated that regulatory T cells (Tregs) were increased because of the increased CD25 expression, but did not confirm Foxp3 co-expression, regulatory cytokine production, nor functional measurements. While Tregs may express CD25, CD25 is also indicative of activated T cells, so whether the elevated levels measured were truly all Tregs or a mixture with activated T cells is unclear. One study evaluated CD4^+^ and γδ T cell responses following infection of pregnant goats (11 weeks of gestation) with wt *B. melitensis* 16M or *B. melitensis* Rev. 1 vaccine (Higgins et al., 2018). They did find a significant increase in the number of IFN-γ-producing γδ T cells 4 weeks post-infection from Rev. 1-infected goats compared to those infected with wt *B. melitensis* 16M.

### B cell immunity and brucellosis

The role of B cells in brucellosis is less understood since induced Abs only modestly or do not protect against *Brucella* challenge (Vitry et al., 2012; de Figueiredo et al., 2015), and B cell-deficient (μMT) mice are found to be more resistant to *Brucella* infection (Goenka et al., 2011; Vitry et al., 2012; Dadelahi et al., 2020). Anti-*Brucella* polysaccharide Abs have less value (Vitry et al., 2012; Boggiatto et al., 2020) than cell-mediated immunity in protection to brucellosis (Godfroid et al., 2005; Goenka et al., 2012; Vitry et al., 2012, 2014; de Figueiredo et al., 2015; Yang et al., 2016; López-Santiago et al., 2019; Wang et al., 2020). Passive transfer of anti-*Brucella* serum was shown to diminish splenic *B. abortus* S19 colonization (Araya et al., 1989), as well as, the passive transfer of an O-polysaccharide-specific mAb for prevention of virulent *B. abortus* in infection (Winter et al., 1989); however, cell-mediated immunity is still required for protection (Jiang and Baldwin, 1993; Zhan and Cheers, 1995; Rodriguez-Zapata et al., 1996; Zhan and Cheers, 1998; Murphy et al., 2001; Ko et al., 2002; Skyberg et al., 2011; Bhagyaraj et al., 2021).

Interestingly, human and animal B cells can be directly infected by *Brucella* (Bratescu et al., 1981; Goenka et al., 2011, 2012; Pesce Viglietti et al., 2016; García-Gil et al., 2019). Consequently, B cells may serve as a reservoir since *Brucella* cannot replicate in B cells (Goenka et al., 2012; Pesce Viglietti et al., 2016), which in turn enables the development of immunosuppressive B cells (Atluri et al., 2011; Goenka et al., 2011, 2012; Spera et al., 2014). Hence, brucellae can persist and sequester in B cells. Further inquiry into B cells’ role in immunity has found that μMT mice were more resistant to *B. abortus* infection than immunocompetent mice (Goenka et al., 2011). Since *B. abortus* can also infect B cells, infection results in the production of B cell-derived TGF-β1, whose anti-inflammatory property may exacerbate chronic *Brucella* infection (Goenka et al., 2012). In fact, adoptive transfer of B and CD4^+^ T cells into Rag1^−/−^ recipients dampened the protective effects of only transferring CD4^+^ T cells against wt *B. melitensis* 16M infection (Dadelahi et al., 2020), further suggesting that infection of B cells leads to suppression of host inflammatory responses (Atluri et al., 2011; Goenka et al., 2011, 2012; Spera et al., 2014).

## *Brucella* vaccines

### Live *Brucella* vaccines

As noted, stimulation of Th1-type immunity derived from CD4^+^ or CD8^+^ T cells is required for protection to eliminate brucellae from the intracellular compartment (Martirosyan et al., 2011; de Figueiredo et al., 2015; Pascual et al., 2018). Given the high DNA homology among *Brucella* species (Wattam et al., 2009; Whatmore and Foster, 2021), vaccination against one species can protect against infection from heterologous *Brucella* species. Current vaccines are limited to four live vaccines used for protecting livestock: *B. abortus* strain 19 (S19) for cattle; rough *B. abortus* RB51 for cattle; *B. melitensis* Rev. 1 for sheep and goats; and *B. suis* strain 2 (S2) for pigs (Olsen and Palmer, 2014; Byndloss and Tsolis, 2016; Goodwin and Pascual, 2016). No vaccines exist for humans, although S19 had been used to vaccinate livestock workers in the former Soviet Union to successfully diminish the incidence of brucellosis (Vershilova, 1961). When the same S19 isolate from P. A. Vershilova along with Rev. 1 were tested in United States volunteers, four of the S19 vaccinees exhibited undesirable sequelae with two recipients being hospitalized; 11 of 16 Rev. 1 vaccinees also showed undesirable sequelae with four of them requiring hospitalization (Spink et al., 1962). A subsequent study using a reduced dose of Rev. 1 still produced some symptoms in vaccinees (Pappagianis et al., 1966).

### *Bacillus abortus* strain 19 vaccine

Derived from a spontaneously attenuated isolate, the *B. abortus* S19 has a 703 base pair deletion of the erythritol catabolic genes (Sangari et al., 1994). S19 was pursued as a cattle vaccine (Buck, 1930), and proved to be effective against brucellosis-induced abortion in cattle (Confer et al., 1985), though its efficacy is only 70% (Lubroth et al., 2007). S19 can also be used therapeutically to reduce incidence of new infections in existing *Brucella*-infected herds (Olsen and Stoffregen, 2005). For susceptible herds, S19 is given to female calves at 3–6 months of age as a single SC dose or to adults at reduced SC or conjunctival dose (Nicoletti, 1990; World Animal Health Organization, 2014). Since S19 still retains its LPS and produces a possible positive serology test, the vaccination regimen for heifers permits protection with a lessened chance of a persistent Ab-reactive response, and also reduces the likelihood of vaccine-induced abortion and vaccine excretion into milk (World Animal Health Organization, 2014).

### *Bacillus abortus* RB51 vaccine

A spontaneous rough mutant was selected after repeated passages of wt *B. abortus* 2308 on rifampin- and penicillin-containing media, and led to the derivation of *B. abortus* RB51 strain (Schurig et al., 1991). RB51’s mutation is due to an interruption of the enzyme, wboA glycosyltransferase, that is involved in O-Ag biosynthesis (Vemulapalli et al., 1999). The advantage of RB51 as a vaccine is its lack of O-Ag (LPS), thus providing a method to distinguish vaccinated from naturally *B. abortus*-infected animals. Since RB51 fails to produce a positive serological reaction by conventional tests, its use allows ease for diagnosis of positive *Brucella* reactivity (Stevens et al., 1994).

Vaccination of cattle with RB51 proved to be safe and efficacious in precluding *Brucella*-induced abortion and fetal infection (Olsen, 2000). Efficacy with RB51 is age-dependent noted by the responsiveness to calf vaccination at >5–6 months of age showing no abortions by pregnant mature cows (2–3 years of age) subjected to *B. abortus* abortion challenge. In contrast, calves vaccinated too young at 3 months of age showed reduced efficacy when challenged as pregnant adults (Cheville et al., 1996; Olsen and Stoffregen, 2005). RB51’s efficacy was found to be similar to S19 (Olsen, 2000). The United states and many other countries have since replaced S19 with RB51 as part of their brucellosis eradication program.

### *Brucella melitensis* Rev. 1 vaccine

Originating from an avirulent streptomycin-dependent strain, *B. melitensis* Rev. 1, was subsequently derived as an isolated revertant that became streptomycin-resistant (Herzberg and Elberg, 1955). Rev. 1 vaccine is administered either as a SC injection or *via* the conjunctiva to sheep and goats, and is protective against virulent *B. melitensis* abortion challenge (Olsen and Stoffregen, 2005). However, Rev. 1 can induce vaccine-induced abortion in pregnant animals, and small ruminants are generally not vaccinated when pregnant (Olsen and Stoffregen, 2005; Byndloss and Tsolis, 2016). Although effective against *B. melitensis*-induced abortion, Rev. 1 being a smooth vaccine makes it difficult to distinguish vaccinated from naturally infected animals. Rev. 1 is also effective in protecting against other *Brucella* species (Byndloss and Tsolis, 2016).

### *Brucella suis* S2 vaccine

*Brucella suis*, responsible for abortion of fetal pigs, is not problematic for United States pork producers since its eradication from commercial herds in 2011, but it does threaten cattle where feral pigs are problematic (Olsen and Tatum, 2016; Franco-Paredes et al., 2017). *Brucella suis* infections of swine remain an international problem (Olsen and Tatum, 2016; Franco-Paredes et al., 2017). The *B. suis* strain 2 (S2) vaccine was developed in China from an isolate taken from an aborted *B. suis*-infected fetal pig, and then subsequently attenuated by repeated passages (Xin, 1986; Zhu et al., 2016). S2 is administered orally *via* drinking water, and has been successfully used in China since 1971 to reduce brucellosis incidence in swine (Xin, 1986; Zhu et al., 2016; Hou et al., 2019). S2 has also been shown to be effective against infection by other *Brucella* species (Xin, 1986; Bosseray and Plommet, 1990; Zhu et al., 2016; Hou et al., 2019).

## Mucosal approaches to brucellosis vaccine delivery

### Advantages of mucosal vaccinations

**Figure 1:**
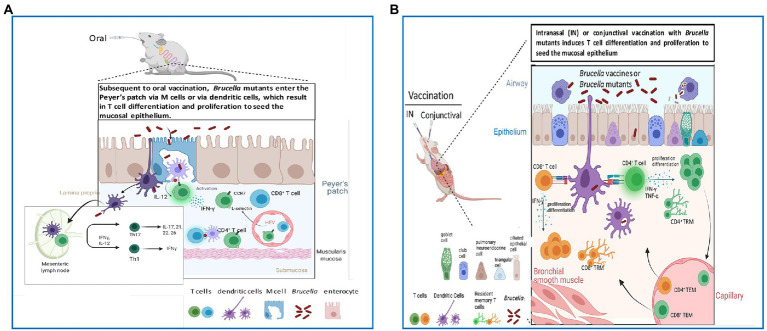
Mucosal routes of vaccination improve host capacity to target T cells to infiltrate the epithelium to combat *Brucella* pathogens. **(A)** Oral vaccination with *Brucella* vaccines or mutant strains enables stimulation of IFN-γ-producing T cells in the Peyer’s patch and eventual dissemination into regional lymph nodes (LNs) and mucosal epithelium. **(B)** Intranasal (IN) and conjunctival vaccination with *Brucella* vaccines or mutant strains enables induction of resident memory T cells (TRMs) to reside in the epithelium to combat reinfection with virulent *Brucella*.

Brucellosis disseminates systemically regardless of the route of exposure (Ko and Splitter, 2003; Atluri et al., 2011; de Figueiredo et al., 2015). A striking advantage of mucosal vaccination is that it arms the mucosa near the sites of infection (Silva-Sanchez and Randall, 2019; Kiyono et al., 2021; Lavelle and Ward, 2022; Mettelman et al., 2022) resulting in the stimulation of memory T cells that prevent reinfection (Hirahara et al., 2021; Lange et al., 2022; Zheng and Wakim, 2022; Figure 1). In addition to stimulating mucosal immunity, mucosal vaccination results in systemic immunity (Silva-Sanchez and Randall, 2019; Kiyono et al., 2021; Lavelle and Ward, 2022; Mettelman et al., 2022). Thus, a key advantage of mucosal vaccination is its capacity to confer protective immunity in both mucosal and systemic tissues. In fact, infection with *Brucella* is the result of a mucosal exposure (Goodwin and Pascual, 2016; Pascual et al., 2018; López-Santiago et al., 2019).

Oral infection with *Brucella* is believed to be mediated *via* the prion protein expressed on intestinal microfold cells (Ackermann et al., 1988; Paixão et al., 2009; Nakato et al., 2012; Figure 1), but brucellae are unable to infect gut tissues (Ablin et al., 1997; Delpino et al., 2007). Rather, infections are believed to be more localized to the lymphoid tissues associated with the naso-oropharyngeal lymph nodes (Meador et al., 1988) resulting from animals sniffing or licking *Brucella*-infected aborted fetuses and/or infected placental tissues (Samartino and Enright, 1993; Schumaker, 2013), and possibly spread through grooming (Rhyan et al., 2019). Similar sites of sensitivity, e.g., tonsils, are also evident in infected humans (Carpenter and Boak, 1932; Poelma and Pickens, 1932; Suraud et al., 2007; Zachou et al., 2008; Ron-Román et al., 2014).

Vaccination *via* naso-oropharyngeal routes offers a potent means for inducing mucosal immunity both locally and at distal immune sites. The nasopharyngeal-associated lymphoid tissue (NALT) and various HNLNs support induction of this process (Csencsits et al., 2002). The HNLNs include the facial or parotid gland LNs (PrLNs); the submandibular gland LNs (SMLNs) also referred to as the superficial cervical LNs; and the deep cervical LNs (CLNs) dorsal to the brachial plexus deep within the musculature of the neck (Tilney, 1971). The SMLNs drain the nasal submucosa, while the CLNs drain the NALT (Tilney, 1971; Hameleers et al., 1990; Kuper et al., 1992). In contrast, the PRLNs are responsible for draining the skin of the head and neck as well as the conjunctiva (Collins, 1978; Freeman and Troutt, 1985; Cserr and Knopf, 1992; Wu et al., 1997; Cashion et al., 1999; Dickstein et al., 1999). It is believed that aerosolization of bacteria infects *via* the conjunctiva (Collins, 1978) since spread of *Salmonella* was prevented in goggled guinea pigs (Moore, 1957). Vaccination *via* the conjunctiva resembles nasal vaccine administration in that similar draining LNs, e.g., SMLNs and PrLNs, are stimulated (Blasco, 1997; Seo et al., 2010). Many of the same regional and distal compartments acquire immune B and T cells noted by the presence of secretory IgA in the tears, saliva, and nasal vaginal washes (Seo et al., 2010). Thus, nasal and conjunctival vaccine delivery impacts cellular immunity presence in the various effector tissues in the head and neck, e.g., naso-oropharyngeal tissues, salivary glands, lungs, and genitourinary tract (Hameleers et al., 1990; Wu et al., 1997; Csencsits et al., 2001, 2002; Seo et al., 2010; Kiyono et al., 2021; Lavelle and Ward, 2022; Mettelman et al., 2022). Given the shared homing signaling among these tissues, vaccination *via* the naso-oropharyngeal and conjunctival tissues can provide regional, systemic, and distal immunity.

Despite the fact that *Brucella* most commonly infects *via* the oral route for both animals and humans, many brucellosis vaccines are parenterally administered both for convenience, and experimentally to mimic systemic disease. Vaccination of a large number of livestock in a short time frame may prove cumbersome and subject the vaccinator to the risk of needle-stick injuries (Buswell et al., 2016; Pereira et al., 2020). The alternative is consideration of mucosal delivery methods. Mucosal vaccinations can circumvent needle-stick injuries since mucosal delivery is needle-free. However, there are limitations as well in mechanizing or implementing mucosal vaccinations for a large number of animals within a limited time period. Nasal vaccinations have the concern of possible draining or sneezing nasal fluids back onto the applicator. Oral vaccination may require gavaging, which may require animal restraint, to administer successfully. Alternatively, food may be mixed with the vaccine (Edmonds et al., 2001; Rhyan et al., 2019) or supplied in the drinking water as done for *B. suis* S2 vaccine, but the latter may not adequately deliver standard doses among the animals (Kiyono et al., 2021).

Oral vaccination has also been tested with S19 to ascertain its capacity for protection against *Brucella*-induced abortion (Nicoletti and Milward, 1983; Nicoletti, 1984). Pregnant heifers in their first trimester were orally vaccinated with 5 × 10^11^ CFUs *B. abortus* S19, and orally challenged with 3.4 × 10^9^ CFUs wt *B. abortus* 2308 at midgestation (Nicoletti and Milward, 1983). Half of all unvaccinated, challenged heifers aborted or had premature delivery and two-thirds were culture-positive while the vaccinated heifers showed only 5% birthing prematurely and 20% being culture-positive (Nicoletti and Milward, 1983). Oral S19 vaccination was found to confer equivalent protection against *B. abortus*-induced abortion challenge to pregnant heifers vaccinated by conventional parenteral or conjunctival routes (Nicoletti, 1984). Oral RB51 vaccination also proved efficacious against *B. abortus*-induced challenge (Elzer et al., 1998). Unbred heifers were orally vaccinated with 5 × 10^10^–1 × 10^11^ CFUs *B. abortus* RB51 mixed with corn syrup on hay for oral consumption, and were bred 6 weeks post-vaccination. All vaccinated and unvaccinated pregnant heifers were challenged by the conjunctival route with 2 × 10^7^ CFUs wt *B. abortus* 2,308. Of the challenged control animals, 80% were culture-positive and 70% aborted compared to RB51-vaccinated animals showing 20% culture-positive and 30% aborted (Elzer et al., 1998). Thus, oral S19 or RB51 conferred equivalent protection to those vaccinated by conventional means (Nicoletti and Milward, 1983; Nicoletti, 1984; Elzer et al., 1998). As shown with *B. suis* S2 vaccine (Xin, 1986), oral delivery of brucellosis vaccines proves to be an effective means for vaccination of livestock and possibly wildlife (Rhyan et al., 2019). To this end, microencapsulated S19 plus sheep liver fluke, *Fasciola hepatica*. Vitelline protein B (VpB), was used to orally vaccinate red deer (Arenas-Gamboa et al., 2009). The VpB protein was included to delay vaccine release from the microspheres. Improved efficacy was observed against conjunctival challenge with 10^9^ CFUs of wt *B. abortus* 2308 compared to red deer vaccinated SC or orally without the VpB. The investigators measured tissue colonization of the spleen, liver, lungs, SMLNs, mammary gland LNs, and mesenteric LNs. These results demonstrate the potential of oral vaccination of ruminants.

### Conjunctival vaccination of livestock

The conjunctiva, or the membrane lining the inner surface of the eyelids, is a surface often susceptible to infection as a result of aerosol dispersion or direct contact with an infectious agent, e.g., *Staphylococcus aureus* and *Chlamydia trachomatis*. The conjunctiva is vascularized and composed of epithelial cells and goblet cells, and has supportive draining lymphatics responsible for immune protection and lubrication for the eyes (Royer et al., 2019). The eyes are considered an immune privileged site (Royer et al., 2019), yet the conjunctiva is often used to vaccinate against brucellosis, especially for goats and sheep (Blasco, 1997; Olsen and Stoffregen, 2005; Olsen and Palmer, 2014). SC Rev. 1 vaccination of sheep and goats during pregnancy results in vaccine-induced abortion, even at a reduced dose (Blasco, 1997; Olsen and Stoffregen, 2005). Too low of a dose results in insufficient protective immunity; hence, conjunctival vaccination was tested and found to reduce the frequency of Rev. 1-induced abortion when using a reduced dose in sheep (Blasco, 1997), but not goats (Zundel et al., 1992). Despite the reduced frequency of vaccine-induced abortion, Rev. 1 administered *via* the conjunctival route can still cause abortion if given to pregnant animals. Reduced abortion frequency was also noted in sheep if vaccinated *via* the conjunctiva during the last month of pregnancy (Jiménez de Bagués et al., 1989). Conjunctival Rev. 1 vaccination also has the advantage of reducing vaccine expression in the milk (Blasco, 1997). Given these outcomes, Rev. 1 vaccination is widely used for vaccination of sheep (Blasco, 1997; Olsen and Stoffregen, 2005).

Conjunctival vaccination with Rev. 1 vaccination of rams limited vaccine infection to the HNLNs and spleen, unlike SC vaccination, which resulted in more generalized infection (Muñoz et al., 2008). Conjunctival vaccination of cattle is not commonly done, but has shown to be beneficial for vaccinating with S19 to elicit lesser enduring serum Ab responses, yet remained protective (Nicoletti, 1990; Chand et al., 2015).

### Mucosal vaccination of experimental animals

Only a limited number of studies have examined the effectiveness of mucosal vaccine approaches, which can induce localized immunity and are more apt to prevent infection. First, to examine the impact of adopting oral vaccination method, one study using orally delivered RB51 proved modestly effective against intraperitoneal (IP; Stevens et al., 1996; Pasquevich et al., 2010) or oral challenge with virulent *B. abortus* 2308 (Stevens et al., 1996). Oral vaccination with irradiated RB51 or *B. neotomae*, a pathogen of wood rats and possibly humans (Suárez-Esquivel et al., 2017), decreased brucellae tissue colonization after IP or nasal challenge with virulent *B. abortus* (Dabral et al., 2014). Examination of various attenuated mutants revealed that oral vaccination with the *ΔpurEK B. melitensis* 16M (WR201) strain elicited robust protection against nasal *B. melitensis* 16M challenge as shown by abating colonization of the lungs and reducing systemic spread (Izadjoo et al., 2004; Yingst et al., 2013). In a similar vein, oral Δ*znuA B. melitensis* vaccination elicited robust protection against pulmonary wt *B. melitensis* 16M challenge, whereby 58 and 83% of the vaccinated mice showed no detectable brucellae in their lungs and spleens, respectively, in an IFN-γ-dependent fashion (Clapp et al., 2016). Interestingly, oral vaccination of IFN-γ^−/−^ mice with the Δ*znuA B. melitensis* mutant elicited even some protection, e.g., reduced tissue colonization by virulent *B. melitensis* 16M, partially, in an IL-17-dependent fashion (Clapp et al., 2011).

Examination of studies adopting nasal delivery methods revealed that nasal immunization with RB51 or a modified RB51 carrying *Brucella* superoxide dismutase (SOD), proved ineffective against pulmonary wt *B. abortus* challenge (Surendran et al., 2011). Prime and boosting failed to augment protection with either RB51 or RB51-SOD. Using a WboA-modified RB51, which produces low amount of cytoplasmic O-polysaccharide, also failed to confer protection against nasal challenge with wt *B. abortus* 2308 (Surendran et al., 2011). However, when RB51WboA was later modified to include overexpression of the *wbkF* gene and referred to as RB51WboAKF strain, increased detectable O-polysaccharide production was capable of eliciting anti-LPS Abs, but still retained its rough phenotype (Dabral et al., 2019). When administered parenterally, it was highly effective in conferring potent protection of the spleen against wt *B. abortus* 2308, wt *B. melitensis* 16M, and wt *B. suis* 1330 challenges (Dabral et al., 2019). The inclusion of soluble TLR2 or TLR4 agonist upon nasal RB51 vaccination enhanced partial protection only in the lungs (Surendran et al., 2013). In contrast, a single, nasal dose of *ΔznuA B. melitensis* potently protected mice against pulmonary *B. melitensis* challenge, wherein more than half of the mice had no detectable brucellae in their lungs or spleens (Clapp et al., 2016). As with the oral vaccinates, protection was IFN-γ-dependent, and the reduced protection in nasally vaccinated IFN-γ^−/−^ mice was abrogated upon IL-17 neutralization (Clapp et al., 2016).

Examination of the types of T cells elicited subsequent to nasal vaccination with *ΔznuA B. melitensis* revealed induction of effector memory CD8^+^ T cells in the lungs producing IFN-γ, TNF-α, and granzyme B with the majority of IFN-γ derived from CD8^+^ T cells (Clapp et al., 2016; Table 1). To ascertain the effectiveness of *B. melitensis* Rev. 1 vaccine and Δ*znuA B. melitensis* mutant to protection relative to contributions by CD4^+^ and CD8^+^ T cells, groups of B6, CD4^−/−^, and CD8^−/−^ mice were nasally vaccinated once, and then nasally challenged 6 weeks later with wt *B. melitensis* 16M. Both B6 and CD4^−/−^ mice showed complete protection in the spleen and lungs by Rev. 1 and Δ*znuA B. melitensis* showing reliance on CD8^+^ T cells (Clapp et al., 2016). Both Rev. 1 and Δ*znuA B. melitensis* exhibited reduced protection in CD8^−/−^ mice. This T cell bias was preserved upon introduction of a second *norD* mutation into Δ*znuA B. abortus* strain. Oral prime, nasal boost of CD8^−/−^ mice with znBAZ resulted in the loss of CD8^+^ T cell-mediated protection against pulmonary challenge with virulent *B. abortus* 2308 (Wang et al., 2020). In contrast, mice vaccinated with znBM-mC depended upon either CD4^+^ or CD8^+^ T cells for protection despite having elevated numbers of CD8^+^ T cells. CD4^−/−^ and CD8^−/−^ mice, orally primed, nasally vaccinated with znBM-mC, showed equivalent protection against virulent pulmonary challenge with wt *B. melitensis* 16M (Goodwin et al., 2022). Such finding indicated that CD4^+^ T cells can compensate for CD8 T cell deficiency.

## Conclusion

Brucellosis is a disease of potential impact to 3.5 billion people (Rossetti et al., 2017), and is considered as a neglected disease (Mableson et al., 2014). Due to its debilitating effects in humans (Pappas et al., 2005, 2006; Franco et al., 2007; Cross et al., 2019), and abortions, reduced fertility, and reduced milk and meat production in livestock (McDermott et al., 2013), this disease poses significant economic hardship to livestock producers in affected countries (World Health Organization, 2005; Rubach et al., 2013; Welburn et al., 2015; Rossetti et al., 2017). Eradication programs used by the United States and some Western countries successfully eliminated and/or controlled livestock brucellosis (Schurig et al., 2002; Olsen and Stoffregen, 2005), but application of such process would prove costly in countries unable to compensate or replace the seropositive livestock (McDermott et al., 2013; Rossetti et al., 2017). Given that current vaccines are only 70% efficacious (Olsen, 2000; Olsen and Stoffregen, 2005; Lubroth et al., 2007), the development of novel vaccines is warranted to help reduce brucellosis incidence. Cheap and readily available vaccines could improve livestock production, improve the standard of living for livestock producers, and reduce incidence of brucellosis in humans.

Most work in brucellosis vaccine development relies on live attenuated mutants. The basis for their employment is their relative success by *B. abortus* S19 and RB51, *B. melitensis* Rev. 1, and *B. suis* S2 vaccines in reducing livestock disease (Olsen and Palmer, 2014; de Figueiredo et al., 2015; Byndloss and Tsolis, 2016; Goodwin and Pascual, 2016). Although these are not completely effective, they provide the basis for successfully using live vaccines to elicit the desired protective response. While not discussed in this article, a subunit vaccine approach could have promise; however, no single *Brucella* protein has been identified yet that is capable of conferring complete protection. Most of the protein candidates are not able to confer more than a two log reduction in tissue colonization (Pascual et al., 2018). More likely, some combination of epitopes is needed to be delivered in such a manner that potent Th1-type responses are elicited.

Consideration of mucosal vaccination delivery methods does need more attention because of their potential impact on immunizing diverse tissues, protecting sites of infection, improving vaccine efficacy, and conferring conventional and alternative mechanisms of protection. Implementing parenteral vaccinations lessens the opportunities for eliciting memory T cell responses at sites of infection that could potentially prevent or limit systemic spread. Several of the studies conducted with ruminants vaccinated or infected with wt *Brucella* did evaluate host T cell responses (Weynants et al., 1998; Suraud et al., 2008; Wang et al., 2014; Dorneles et al., 2015; Curina et al., 2018; Higgins et al., 2018; Boggiatto et al., 2020), but many of these studies limited evaluations to peripheral blood T cells, and not mucosal T cells. Peripheral blood T cells vary in their stage of activation, and are in transition in homing to their targeted tissues. Aside from looking at peripheral blood, T cell restimulation methods may also influence Ag-specific responses. Among these studies, the described restimulation methods varied, where often whole LPS-bearing brucellae were used. LPS can alter host T cell responses *via* activation of co-cultured Ag-presenting cells (APCs). Finally, mode of Ag restimulation may bias T cell responses where soluble Ags mostly stimulate *via* the MHC class II-dependent pathway activating CD4^+^ T cells, and in contrast, an infection process, e.g., live infection of APCs, followed by subsequent APC inactivation, promotes MHC class I-driven CD8^+^ T cell responses. Future studies are warranted to compare the influence of the route of delivery, e.g., SC, conjunctival, oral, and nasal, upon eliciting memory T cell responses, especially the naso-oropharyngeal tissues of animals and humans, which are sensitive to *Brucella* infection (Carpenter and Boak, 1932; Poelma and Pickens, 1932; Suraud et al., 2007, 2008; Zachou et al., 2008; Arenas-Gamboa et al., 2009; Ron-Román et al., 2014; von Bargen et al., 2015; Rouzic et al., 2021).

Proof of mucosal vaccination effectiveness has been shown experimentally in livestock, wildlife, and rodents. Oral vaccination with S19 (Nicoletti and Milward, 1983; Nicoletti, 1984) and RB51 (Elzer et al., 1998) proved effective in pregnant heifers against *B. abortus*-induced abortion. Oral vaccination of red deer with microencapsulated S19 effectively reduced tissue colonization (Arenas-Gamboa et al., 2009). Conjunctival Rev. 1 vaccination of sheep proved effective in protection against abortion and vaccine-induced abortion (Jiménez de Bagués et al., 1989; Blasco, 1997). Conjunctival brucellosis vaccination is commonly practiced (Blasco, 1997; Chand et al., 2015), but understanding why this route is effective in livestock is less understood. Given difficulties in conducting immune analyses in livestock, experimental animal systems can shed insight into mechanisms of protection not otherwise possible in livestock. Using the Δ*znuA B. melitensis* mutant (Clapp et al., 2011, 2016), znBAZ mutant (Wang et al., 2020), znBM-mC mutant (Goodwin et al., 2022), Rev. 1 vaccine (Clapp et al., 2016), and Δ*purEK B. melitensis* (WR201) mutant (Izadjoo et al., 2004; Yingst et al., 2013), studies proved that potent protection can be achieved in the mucosal and systemic compartments. An interesting finding derived from the Δ*znuA B. melitensis* (Clapp et al., 2011, 2016), znBAZ (Wang et al., 2020), and znBM-mC (Goodwin et al., 2022) studies was the predilection for stimulation of IFN-γ^+^ CD8^+^ T cell responses, which is attributed to either the mutant or mode of delivery. IP vaccination with znBAZ stimulated both CD4^+^ and CD8^+^ T cells, but the number of splenic IFN-γ^+^ CD4^+^ T cells nearly doubled those induced by CD8^+^ T cells (Yang et al., 2016) suggesting that the route has an influence. This was particularly noted with nasal Rev. 1 vaccination resulting CD8^+^ T cell-dependent immunity as observed by the reduction in protection in CD8^−/−^ mice (Clapp et al., 2016). However, oral vaccination with the WR201 mutant induced CD4^+^ T cell-dependent protection since protection against nasal challenge with wt *B. melitensis* 16M were equivalent in B6 and CD8^−/−^ mice (Yingst et al., 2013). This finding disputes the idea that route of vaccination is the only factor, but the mutant is also an important consideration. These collective findings demonstrate that immune protection can be achieved with either CD4^+^ or CD8^+^ T cells, but eliciting memory responses proximal the site of infection, e.g., the stimulation of TRMs, are more apt to protect, possibly preventing or limiting brucellae dissemination as suggested by the Δ*znuA B. melitensis* (Clapp et al., 2016), znBAZ (Wang et al., 2020), and znBM-mC (Goodwin et al., 2022) studies. Mucosal vaccination with znBAZ or znBM-mC stimulated robust CD8^+^ and CD4^+^ TRM responses in the lungs to facilitate protection against pulmonary wt challenge, and may have prevented further brucellae dissemination (Wang et al., 2020; Goodwin et al., 2022). Thus, the stimulation of alternative immune pathways is beneficial, and may improve vaccine efficacy warranting further testing.

The study of different *Brucella* species may assist in learning *Brucella*’s pathogenesis and tenets of host protection. The discovery of *B. microti* in voles (Scholz et al., 2008) and later in other host species (Occhialini et al., 2022), extends the genus diversity, whereby *B. microti* shares metabolic traits, conserved VirB type IV secretion system, and early granuloma formation with other *Brucella* pathogens of livestock (Scholz et al., 2008; Hanna et al., 2011; Jiménez de Bagüés et al., 2011; Occhialini et al., 2022). *Brucella microti* offers another model to study brucellosis since it can be lethal in mice and actively proliferates in murine macrophages. Cell-mediated immunity is essential for protection against *B. microti* and NK cells are required (Jiménez de Bagüés et al., 2011) showing the relevance of experimental animal systems.

Finally, an improved vaccine for one species has the potential to cross-protect against other *Brucella* species. A number of studies have shown such cross-protection as evidenced in humans with S19 for protection against *B. melitensis* infection (Vershilova, 1961; van Straten et al., 2016); *B. neotomae* for protection against *B. abortus* (Dabral et al., 2014); S19, Rev. 1, and S2 for cross-protection against *B. suis, B. melitensis*, and *B. abortus* (Bosseray and Plommet, 1990); and RB51WboAKF for *B. suis, B. melitensis*, and *B. abortus* (Dabral et al., 2019). Herein lies the potential for a universal brucellosis vaccine.

## Author contributions

DP conceived and designed the entire review and wrote the paper. ZG, EB, CH, and XY reviewed and edited the manuscript. ZG, EB and DP constructed the figures. All authors contributed to the article and approved the submitted version.

## Funding

This work was supported by U. S. Public Health Grants R01 AI123244 and R01 AI125546, and ZG was in part supported by AI123244-S1.

## Conflict of interest

The authors declare that the research was conducted in the absence of any commercial or financial relationships that could be construed as a potential conflict of interest.

## Publisher’s note

All claims expressed in this article are solely those of the authors and do not necessarily represent those of their affiliated organizations, or those of the publisher, the editors and the reviewers. Any product that may be evaluated in this article, or claim that may be made by its manufacturer, is not guaranteed or endorsed by the publisher.
